# Implantable, Bioresorbable Radio Frequency Resonant Circuits for Magnetic Resonance Imaging

**DOI:** 10.1002/advs.202301232

**Published:** 2023-06-25

**Authors:** Geumbee Lee, Mark D. Does, Raudel Avila, Juyeon Kang, Kevin D. Harkins, Yunyun Wu, William E. Banks, Minsu Park, Di Lu, Xinqiang Yan, Jong Uk Kim, Sang Min Won, Adam G. Evans, Jeremy T. Joseph, Christopher L. Kalmar, Alonda C. Pollins, Huseyin Karagoz, Wesley P. Thayer, Yonggang Huang, John A. Rogers

**Affiliations:** ^1^ Querrey Simpson Institute for Bioelectronics Northwestern University Evanston IL 60208 USA; ^2^ Department of Biomedical Engineering Vanderbilt University Nashville TN 37235 USA; ^3^ Vanderbilt University Institute of Imaging Science Vanderbilt University Medical Center Nashville TN 37232 USA; ^4^ Department of Mechanical Engineering Northwestern University Evanston IL 60208 USA; ^5^ Department of Chemical and Biomolecular Engineering Korea Advanced Institute of Science and Technology Daejeon 34141 Republic of Korea; ^6^ Department of Radiology and Radiological Sciences Vanderbilt University Medical Center Nashville TN 37232 USA; ^7^ School of Microelectronics University of Science and Technology of China Hefei Anhui 230026 China; ^8^ Department of Electrical and Computer Engineering Sungkyunkwan University 2066, Seobu‐ro, Jangan‐gu Suwon‐si Gyeonggi‐do 16419 Republic of Korea; ^9^ Department of Plastic Surgery Vanderbilt University Medical Center Nashville TN 37232 USA; ^10^ Department of Materials Science and Engineering Department of Biomedical Engineering Department of Neurological Surgery Northwestern University Evanston IL 60208 USA

**Keywords:** biomedical implants, bioresorbable devices, LC‐resonant circuits, magnetic resonance imaging, radiofrequency coils

## Abstract

Magnetic resonance imaging (MRI) is widely used in clinical care and medical research. The signal‐to‐noise ratio (SNR) in the measurement affects parameters that determine the diagnostic value of the image, such as the spatial resolution, contrast, and scan time. Surgically implanted radiofrequency coils can increase SNR of subsequent MRI studies of adjacent tissues. The resulting benefits in SNR are, however, balanced by significant risks associated with surgically removing these coils or with leaving them in place permanently. As an alternative, here the authors report classes of implantable inductor–capacitor circuits made entirely of bioresorbable organic and inorganic materials. Engineering choices for the designs of an inductor and a capacitor provide the ability to select the resonant frequency of the devices to meet MRI specifications (e.g., 200 MHz at 4.7 T MRI). Such devices enhance the SNR and improve the associated imaging capabilities. These simple, small bioelectronic systems function over clinically relevant time frames (up to 1 month) at physiological conditions and then disappear completely by natural mechanisms of bioresorption, thereby eliminating the need for surgical extraction. Imaging demonstrations in a nerve phantom and a human cadaver suggest that this technology has broad potential for post‐surgical monitoring/evaluation of recovery processes.

## Graphical abstract

A bioresorbable, implantable radio frequency electronic device enhances key capabilities in magnetic resonance imaging (MRI). These simple, small bioelectronic systems function over clinically relevant time frames at physiological conditions and then disappear completely by natural mechanisms of bioresorption, thereby eliminating the need for surgical extraction. Imaging demonstrations suggest that this technology has broad potential for post‐surgical monitoring/evaluation of recovery processes.

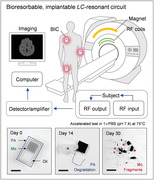

## 1 Introduction

Magnetic resonance imaging (MRI) is a powerful methodology with contrast that can be made sensitive to a myriad of physical, chemical, and functional characteristics of tissue. Harnessing these sensitivities to provide quantitative and specific imaging of biomarkers is a central idea of many MRI research programs. Often, the critical barrier to progress in the development of quantitative MRI (qMRI) methods is the signal‐to‐noise ratio (SNR). Approaches for increasing SNR include increasing the static magnetic field strength (*B*_0_), extending the scan duration, and lowering the spatial resolution.^[^ ^1^, ^2^ ^]^ The SNR increases, however, only approximately linearly with *B*_0_. MRI systems typically use *B*_0_ of 0.1–9.4 Tesla (T); 4.7 T systems used this work allow small animal imaging.^[^ ^3^ ^]^ Likewise, the SNR improves only with the square root of scan duration and is ultimately limited by patient comfort and/or motion. Decreasing the resolution limits the sizes of structures or regions that can be investigated.

Alternatively, image SNR can be increased by using a small, locally targeted radiofrequency (RF) coils to receive the MR signal. The original application of such coils uses placement on the surface of the skin to increase the SNR in MR spectroscopy studies.^[^ ^4^ ^]^ Current clinical MRI systems include an assortment of receive coils with sizes and shapes designed for specific body parts (e.g., head, knee, spine, wrist, and so on). Similarly, endoscopic coils can reach some deep tissues, such as the prostate.^[^ ^5^ ^]^ Some pre‐clinical animal studies use coils surgically implanted directly onto or adjacent to tissues of interest, with wireless connection via inductive coupling.^[^ ^6^, ^7^, ^8^ ^]^ Experimental studies,^[^ ^9^, ^10^, ^11^ ^]^ and theoretical calculations^[^ ^10^, ^11^ ^]^ indicate that this approach can increase the SNR by several fold. A similar mode of use could apply to humans, where the coil serves as a temporary implant, surgically implanted before a specific diagnostic need and extracted after. For example, an important potential application is in tracking the extent of axonal regeneration after the surgical repair of a peripheral nerve injury. An RF coil placed adjacent to the nerve and distal to the repair site could provide high SNR MRI to quantitatively evaluate the extent of regeneration prior to clinical signs of recovery.^[^ ^12^, ^13^ ^]^

Prohibitive disadvantages of this approach follow, however, from the costs and patient risks associated with surgical extraction of the implanted coil. Of relevance in this context is an emerging area of materials science that enables electronic devices that are fully bioresorbable, with demonstrated examples in intracranial pressure^[^ ^14^, ^15^ ^]^ and temperature^[^ ^16^ ^]^ monitors, neuroregenerative nerve stimulators,^[^ ^17^ ^]^ cardiac pacemakers^[^ ^18^ ^]^ and systems for spatio‐temporal mapping of electrophysiological activity across the surface of the brain.^[^ ^19^ ^]^ These and other related technologies support stable operation in the body over time periods of interest (days to weeks), and then completely disappear in a harmless manner through the processes of bioresorption into surrounding biofluids, typically via metabolic action or hydrolysis. These mechanisms eliminate device load and associated risks to the patient without the need for surgical extraction and on timescales that meet clinical requirements.

Here, we present a fully bioresorbable, implantable RF resonant circuit, referred to in the following as a coil (bioresorbable implantable coil, BIC), that improves the SNR in MRI by its proximity to a tissue of interest. Careful choices in materials and design layouts enable mechanically flexible, compact, and lightweight forms, with ability to support stable operation during a desired timeframe. Bioresorbable metals such as magnesium (Mg), molybdenum (Mo), zinc (Zn) or tungsten (W) define coils that reside in bioresorbable enclosures formed using a polyanhydride (PA)‐based polymer and filled with an edible oil as a barrier to penetration of surrounding biofluids. This material design minimizes unwanted drift in the RF properties for at least 30 days in phosphate buffered saline (PBS; pH 7.4) at body temperature, with the capability to support high SNR in MRI. Experimental demonstrations described in this work use such devices as implants to enhance imaging of phantoms and of a human cadaver arm following a surgical intervention, with comparisons to results obtained using standard, non‐bioresorbable devices based on copper coils.

## 2 Result and Discussion

**Figure 1:**
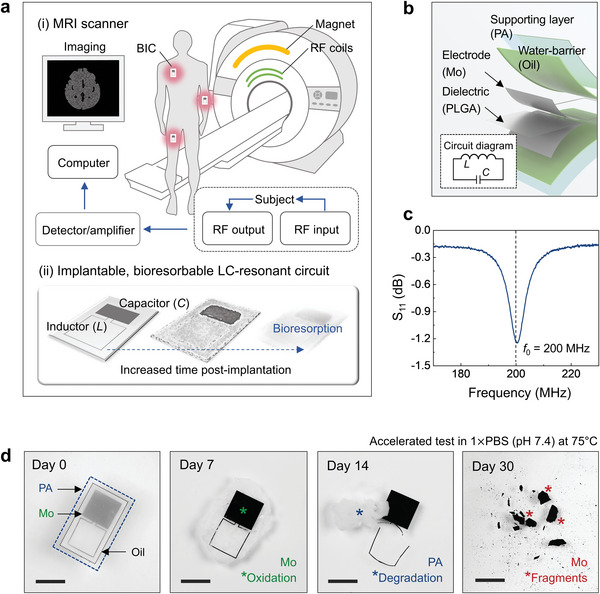
Implantable, bioresorbable resonant circuits for enhanced magnetic resonance imaging. a) Schematic illustrations of a conventional MRI scanner operated with a human subject that has an implant (top), and the process of bioresorption of the device post‐surgery (bottom). b) Exploded‐view illustration of the materials and design features of the device, which consists of a single inductor (square layout; 1 turn, 7 mm diameter, 200 µm trace width) and a single capacitor (≈42 mm^2^ area for each electrode), encapsulated in a structure of PA and edible oil. Inset, circuit diagram. c) Measured RF behavior (S_11_) of the device. The resonant frequency (*f*_0_) is ≈200 MHz. d) Sequence of images that shows accelerated dissolution of a device during immersion in 1 × PBS (pH 7.4) at 75 °C for 30 days. Scale bars, 10 mm.

MRI is a non‐invasive, three–dimensional (3D) diagnostic imaging tool that primarily visualizes water and fat, making it particularly well suited to imaging soft tissue. A traditional MRI scanner consists of a large magnet, RF coils (transmitter and receiver), and a computer that controls the operation of a supporting collection of electronics (**Figure** **1**a, top). The human body is ≈60% water by mass, and each water molecule includes two hydrogen atoms. The nucleus of a hydrogen atom (a proton) has properties of a magnetic moment and angular momentum. In a large external magnetic field (*B*_0_), these magnetic moments tend to align and result in a net magnetization per unit volume of tissue. During an MRI exam, RF pulses perturb the magnetization from equilibrium resulting in magnetization that precesses at rates determined by *B*_0_ according to the Larmor equation: *ω*_0_ = *γB*_0_/2*π* (*ω*_0_, the precession frequency; *B*_0_, the static magnetic field; and γ, gyromagnetic ratio of the nucleus), corresponding to *ω*_0_≈ 128 MHz for *B*_0_ = 3 T, a typical clinical MRI field strength. A near‐field antenna (i.e., RF receiver coil) detects the resulting fluctuating magnetic field from the precessing magnetization.^[^ ^20^, ^21^ ^]^ A key limitation is that the net magnetization is quite small. Consequently, the signal detected by the RF coil can be similar in amplitude to the root‐mean‐squared thermal noise signal, thereby leading to a small SNR. The SNR is proportional to the volume of imaging volume element (voxel), and thus decreases with increasing spatial resolution. The image SNR is a particularly important limiting factor to quantitative MRI (qMRI) methods, which typically involve voxel‐wise analysis of the MRI signal intensity from a series of images with varied contrast. Accurate and precise fitting of such MRI intensities to a signal or tissue model generally demands high SNR.

The bottom schematic illustration in Figure 1a presents an envisioned scenario for clinical use of a BIC designed to improve the SNR throughout a recovery period following a surgery, but ultimately to dissolve away in surrounding biofluids in a natural and harmless manner. The device includes an *LC*‐resonant circuit, consisting of one inductor (*L*) and one capacitor (*C*) (Figure 1b). For the example reported here, the inductor is a single conductive loop (square layout; 1 turn, 7 mm diameter, 200 µm trace width) defined by a 50 µm thick trace of Mo. The capacitor consists of a pair of Mo plates (50 µm thick and ≈42 mm^2^ area for each) separated by an insulating film of a poly(lactide‐*co*‐glycolide) (PLGA; 65:35 [lactide:glycolide], 35 µm thick). The PLGA bonds to the Mo with a bioresorbable adhesive (polyvinyl alcohol [PVA]) coated on the plates via hot‐pressing (≈70 °C, close to glass transition temperature both PLGA and PVA). A laser ablation process patterns the Mo features (i.e., the loop and the plates) from a uniform foil. In addition to using Mo and PLGA, other bioresorbable metals and dielectric materials with relatively high conductivity and permittivity can be considered. An encapsulating structure of PA surrounds the circuit. Edible oil (i.e., palm kernel oil) fills the interior region between the inner walls of this structure and the circuit, as a barrier to penetration of biofluids. The presence of this oil minimizes drifts in the resonant frequency that can result from diffusion of water through the PA, leading to partial hydrolysis of the Mo. This bilayer (inner layer: oil; outer layer: PA) is of interest due to its good water barrier properties,^[^ ^22^, ^23^ ^]^ along with its improved mechanical flexibility and physical toughness under body temperature by comparison to alternatives such as natural wax materials (Figure S1, Supporting Information).^[^ ^24^, ^25^ ^]^ In situ photopolymerization of PA allows for complete embedding the oil‐encapsulated BIC into a polymeric enclosure, thereby minimizing concerns about the robustness of the interface between two layers. The overall device is small (12 mm wide, 14 mm long), thin (<700 µm thick), lightweight (≈0.2 g) and flexible, thereby facilitating surgical implantation and minimizing mechanical loads on surrounding soft tissues. Details of the fabrication procedures and device dimensions are in the Experimental Section and Figure S2, Supporting Information.

**Figure 2:**
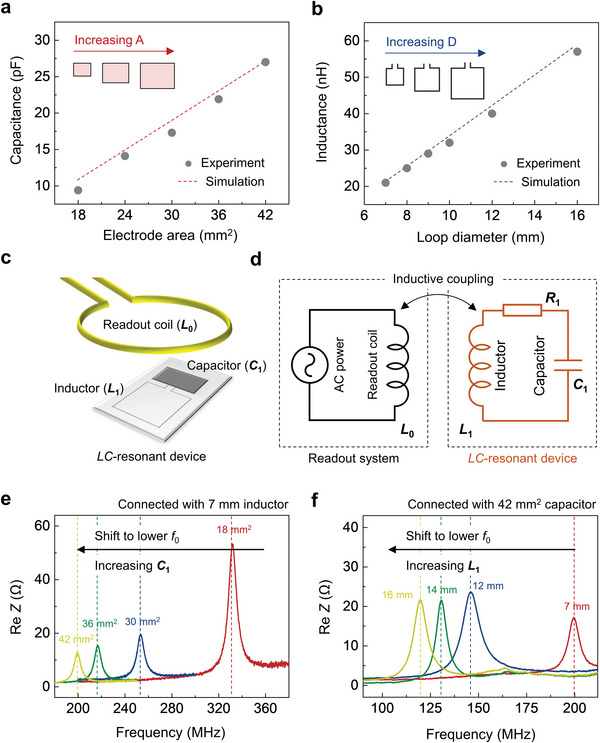
Design features and associated electromagnetic properties of bioresorbable *LC*‐resonant circuits. a) Experimental (dots) and simulated (dash lines) results for the dependence of the capacitance on the area of the plate and b) for the dependence of the inductance on the loop diameter. c,d) Schematic illustration (left) and equivalent circuit diagram (right) of an experimental setup to determine the *f*_0_ of the *LC*‐resonant circuits. *L*_0_: inductance of the readout coil; *L*_1_: inductance of the device coil (square layout, 1 turn); *C*_1_: capacitance of the parallel‐plate capacitor (metal/insulator/metal structure). Near‐field magnetic coupling between the device coil and the readout coil and enables determination of *f*_0_ through measurements of the impedance. e,f) Measured changes in the real part of the impedance (Re *Z*) for *LC*‐resonant circuits with different designs. The *f*_0_ shifts toward lower frequency with increases in the electrode area of the capacitor (i.e., higher capacitance) and the loop diameter of the inductor (i.e., higher inductance).

This *LC*‐device resonates at a frequency, *f*_0_, defined by the peak of the real part of the impedance. For cases presented here, *f*_0_ is ≈200 MHz (Figure 1c, capacitance ≈30 pF; inductance ≈21 nH), corresponding to the proton Larmor frequency at 4.7 T MRI (e.g., ≈64 MHz at 1.5 T MRI; ≈128 MHz at 3 T MRI).^[^ ^26^ ^]^ Figure S3, Supporting Information, summarizes the RF properties as a function of the thickness of the Mo electrode. Although the *f*_0_ is independent of thickness, the power transfer efficiency decreases with the thickness, from 50 to 15 µm. Other operating parameters are possible through modifications in the designs of the inductor and capacitor. For additional experimental results, refer to **Figure** **2**.

The constituent materials are bioresorbable by hydrolysis/dissolution due to interactions with surrounding biofluids under physiological conditions, as demonstrated in previous studies. For example, PVA is a biocompatible polymer that dissolves within a few minutes (molecular weight dependent) upon immersion in water.^[^ ^27^, ^28^, ^29^ ^]^ Multiple hydroxyl groups (—OH) in PVA allow water molecules to permeate into the polymer chain network, resulting in swelling and dissolution. PLGA degrades into nutritious compounds, lactic acid and glycolic acid, with different dissolution rates depending on the ratio of lactide to glycolide in the PLGA (e.g., ≈1.2 µm d^−1^ for 50:50 ratio).^[^ ^30^ ^]^ The oil used here mainly consists of fatty acids that are biocompatible and edible,^[^ ^31^, ^32^ ^]^ capable of metabolic breakdown and resorption by the body. The form of bioresorbable PA used here has a dissolution rate of ≈1.3 µm d^−1^.^[^ ^33^ ^]^ Mo undergoes hydrolysis to yield a corresponding water‐soluble acid, 2Mo + 2H_2_O + 3O_2_ → 2H_2_MoO_4_.^[^ ^34^ ^]^ The projected time for full dissolution of Mo structures with thicknesses of 50 µm, based on a previously published rate of 0.02 µm d^−1^ at physiological conditions,^[^ ^35^ ^]^ is several years. Accelerated aging tests in 1  × PBS (pH 7.4) at elevated temperatures reveal the kinetics of these processes. Figure 1d shows the dissolution behavior of a complete device at 75 °C (≈16 times relative to 37 °C).^[^ ^36^ ^]^ The use of different molar ratios of PA and of thinner Mo electrodes (15 µm thick) leads to accelerated degradation of entire platform. The PA degrades first within 7 days, followed by exposure of the oil and Mo electrodes to water. The Mo gradually undergoes oxidation and breaks into several pieces with loss of the PA substrate within 14 days. Finally, the Mo disintegrates into fine fragments after 30 days, and ultimately dissolves completely.

The *B*_0_ for MRI defines the necessary values for *f*_0_, which is related to the capacitance of the parallel plate capacitor and the inductance of the coil, according to $f_{0}=\;1/2\pi \sqrt{LC}$. The capacitance depends on the areas of overlap between the plates, their separation, and the permittivity of the dielectric layer, consistent with the results in Figure 2a for the case of a 35 µm thick layer of PLGA as the dielectric. The inductance depends on the parameters of the coil, such as the number of turns, the loop diameter, the wire diameter, and the relative permeability of the surroundings. The inductance in such cases increases monotonically with the diameter, as demonstrated experimentally and analytically in Figure 2b (see Figure S4, Supporting Information, for simulated summaries of device characteristics as a function of frequency by finite element analysis [FEA]). Figure 2c,d presents a diagram of the setup for determining *f*_0_. Near‐field magnetic coupling between the device coil (inductance, *L*_1_) and a readout coil (inductance, *L*_0_) enables measurements of the impedance (*Z*) as a function of frequency (*f*) across *L*_0_. The peak of the real part of the impedance (Re *Z*) defines *f*_0_, through fits of measurements of the real and imaginary parts of the S‐matrix (S_11_) deduced from the equivalent circuit and the formula outlined in the Experimental Section. Changes in *f*_0_ with these various inductance and capacitance values demonstrate the tunability of the *LC*‐resonant circuit, consistent with expectation as illustrated in Figure 2e,f. Reductions in the overall size of the device can facilitate surgical implantation. For instance, modifying the layout between a loop and capacitor or reducing the thickness of the dielectric layer represent two options for miniaturizing these devices (Figure S5, Supporting Information).

When designing such devices, a key consideration is aligning *f*_0_ to that of the MRI instrument. As an implant, the BIC should maintain a value of *f*_0_ close to the design point, in the cases reported here ≈200 MHz, over a clinically relevant time frame. As mentioned previously, oil (inner layer) is attractive because of its hydrophobic and water‐resistant character. The bioresorbable, photocurable version of PA (outer layer) used here mechanically supports the oil‐encapsulated device during implantation. At body temperature, the oil becomes a quasi‐solid due to its melting point (*T*_m_, ≈37–39 °C), thereby providing enhanced flexibility to the entire platform (Figure S1, Supporting Information). Figure S6, Supporting Information and the Experimental Section describe details associated with the encapsulation procedures.

**Figure 3:**
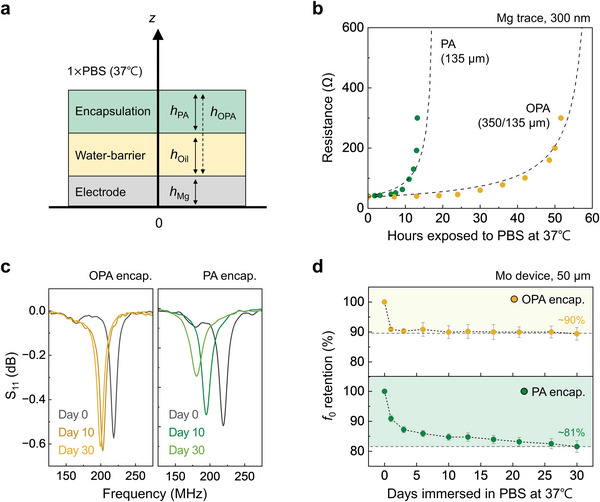
Characteristics of encapsulating materials, in isolation and as used in the BIC. a) Schematic illustration of a reactive diffusion model for the OPA encapsulating strategy applied to an electrode test structure. b) Water permeability of layers of OPA and PA. The measurements show changes in the theoretical (dash lines) and measured (dots) resistances of Mg thin traces (≈300 nm thick) encapsulated with PA (135 µm thick) and OPA (oil: 350 µm thick, PA: 135 µm thick) layers during exposure to 1 × PBS (pH 7.4) at 37 °C. c) Measured RF behavior (S_11_) and d) drifts in the *f*_0_ of the BIC encapsulated with OPA (top, yellow) and PA (bottom, green) during immersion in 1 × PBS (pH 7.4) at 37 °C. Independent samples, *n* = 4. All error bars, standard deviation.

Impermeability to water is an important property for the encapsulating layers in bioresorbable electronic devices because these materials affect the functional lifetime of the electronic components. The PA used here has a hydrophobic character and resulting surface eroding mechanism for bioresorption.^[^ ^33^ ^]^ Because this polymer is somewhat permeable to water, however, a layer of oil must be added to provide a sufficient barrier to water penetration for the application considered here. Studies of relative values of permeability for single (i.e., PA) and double (i.e., oil/PA; OPA) layer encapsulating structures reveal the effects. A 1D analytical model of reactive diffusion (Note S1, Supporting Information)^[^ ^37^ ^]^ captures the kinetics of water permeation with the OPA encapsulation (**Figure** **3**a). The water diffusivities (*D*) of PA and oil layers estimated in this manner are *D*_PA_ = 0.97 ×10^−13^ and *D*_OPA_ = 2.6 × 10^−15^ m^2^ s^−1^, respectively. *D*_PA_ is comparable to the lower bound of reported values for bioresorbable polymers, and *D*_OPA_ is significantly lower than that of most other materials options.^[^ ^24^, ^38^ ^]^ This model suggests that a layer of oil layer can effectively improve the water‐barrier properties compared to strategies that use only the PA layer.

Figure 3b summarizes changes in the resistance of test structures that consist of serpentine Mg traces (300 nm thick) patterned on silicon dioxide substrates and encapsulated with PA (135 µm thick) and OPA (oil: 350 µm thick, PA: 135 µm thick) upon exposure to 1 ×PBS (pH 7.4 at 37 °C). Gradual dissolution of thin films of Mg can be exploited to define the relative water permeability of layers of material cast on top (see Figure S7, Supporting Information, for the experimental setup). Water can penetrate the PA, thereby leading to substantial dissolution of the Mg and corresponding dramatic increases in the resistance within 12 h. By contrast, traces encapsulated in OPA show little change in resistance for 50 h. The rate of change in resistance, in a percentage sense, can be controlled by the thicknesses of the encapsulation layers and the metal.^[^ ^33^ ^]^ Figure S8, Supporting Information, shows changes in the resistance of Mg foils (20 µm thick, same design with thin films), encapsulated by PA and OPA. The resistances remain largely invariant for tens of days and the Mg foil encapsulated with an OPA layer exhibits improvements in lifetime (corresponds to the time that electrical resistance maintains finite) that are consistent with tests using the Mg film (300 nm thick).

Biofluid permeation can lead to variations in *f*_0_ for *LC*‐resonant devices, due to mechanisms that involve both changes in the dielectric properties (i.e., permittivity of hydrated polymers)^[^ ^39^ ^]^ and the conductivities (i.e., oxidation in bioresorbable conductors) of the materials.^[^ ^40^ ^]^

The Experimental Section and Figure S6, Supporting Information, illustrate the process for forming encapsulating layers to slow these processes. A mold guides the delivery of a liquid mixture of PA by capillary action through an inlet to an opposite outlet. Passing ultraviolet light through the transparent mold enables photopolymerization of PA to embed a device pre‐coated with a layer of oil in a solid structure. Pre‐coating of the device with the oil follows the same molding procedure but using molten oil instead of liquid PA. Molten oil infills the mold and cooling yields its solidification, resulting in a fully oil‐encapsulated device. The geometries of the molds define the key dimensions.

The gradual diffusion of water, whose dielectric constant is large at these frequencies (*ε* ≈75 at 37 °C), into the PA (*ε* ≈3–4), can increase the capacitance of the device, thereby steadily reducing *f*_0_ (Figure 3c,d; Figure S9, Supporting Information).^[^ ^41^ ^]^ Observations during soak tests that extend to 30 days indicate that *f*_0_ of the OPA‐encapsulated device shifts for a certain period of time (1–2 days on average) after immersion in 1 × PBS (pH 7.4 at 37 °C) and then stabilizes to an invariant value (Figure 3c,d). As a result, pre‐immersing OPA devices with a *f*_0_ of ≈220 MHz into 1  × PBS (pH 7.4 at 37 °C) for several days stabilizes the frequency at ≈200 MHz and prepares them for implantation (Figure S10, Supporting information). Drying the hydrated devices returns *f*_0_ to its original value (Figure S11, Supporting Information), for cases when oxidation and hydrolysis of the metal features can be neglected.

**Figure 4:**
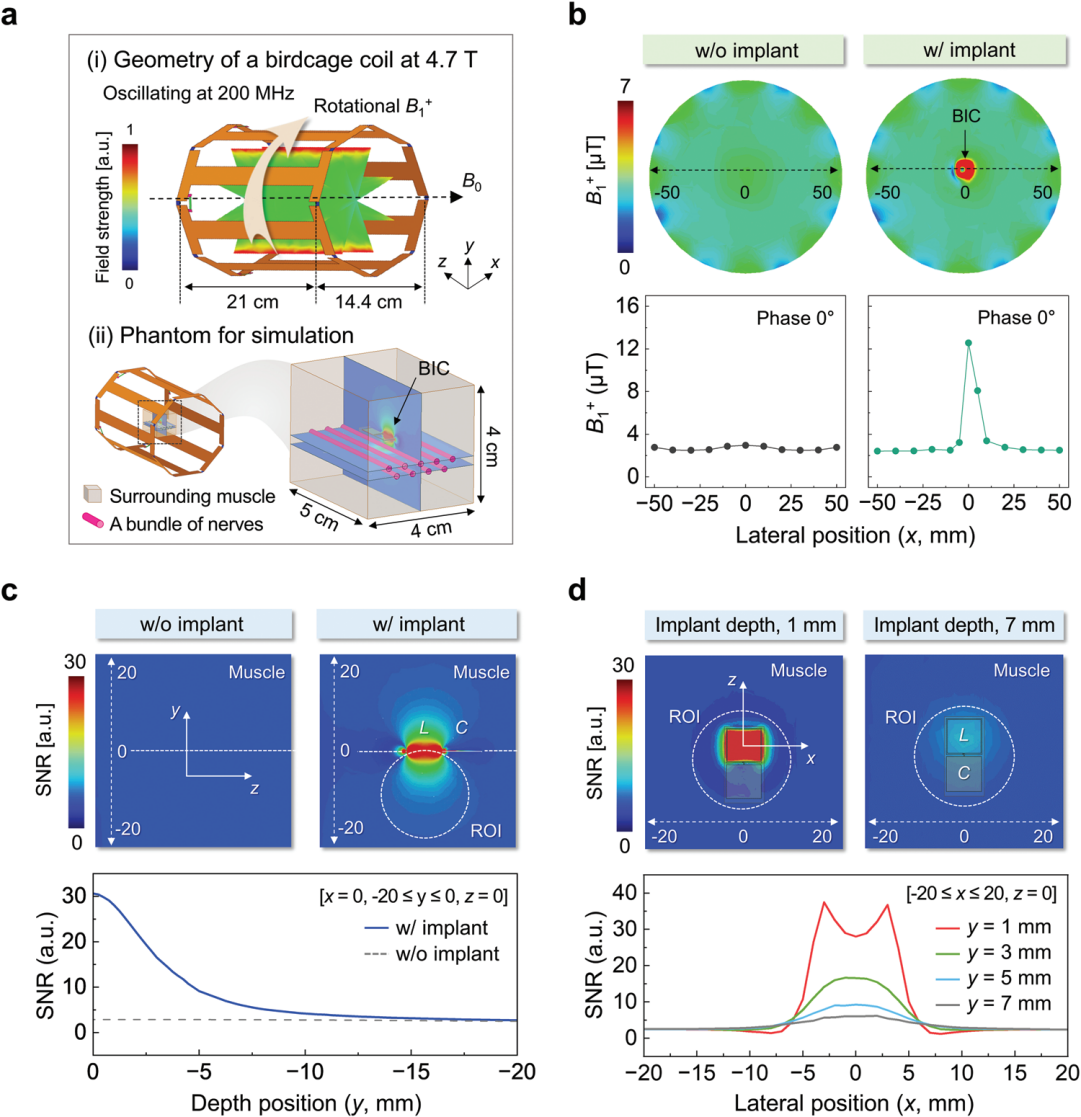
Simulations of image enhancement with a BIC designed to operate with an MRI scanner at 4.7 T. a) Schematic illustration of the specifications of a birdcage (top) and a phantom (bottom) used in the simulations. b) Simulated distribution of *B*_1_^+^ for a case without and with a BIC. c) Simulated spatial maps of SNR along the *y*‐direction for different depth positions. A position far from the BIC results in a decrease in SNR. ROI, circle with a radius of 10 mm. d) Simulated SNR profiles along the *x*‐direction for various depths of implantation of the BIC. Deep implantation results in a decrease in SNR. ROI, circle with a radius of 10 mm.

FEA quantifies the influence of the BIC on the electromagnetic fields and signal amplitudes for simulated imaging cases. The modeling features an eight‐element low‐pass birdcage coil (an RF‐transmit coil in MRI with two end rings and multiple legs and capacitors) that generates a homogeneous and circularly polarized magnetic field *B*_1_, perpendicular to the direction of the stationary field *B*_0_, based on a sinusoidal current distribution in the legs of the cage at the dominant resonance mode of the RF structure.^[^ ^42^, ^43^ ^]^ **Figure** **4**a (top) and Figure S12, Supporting Information, show the geometry and dimensions of the birdcage coil at 4.7 T. The model includes a phantom with cylindrical inclusions (1 mm diameter) to approximate a tissue section of muscle with nerve bundles, using material properties listed in Table S1, Supporting Information. The BIC resides at the center of the cage (0, 0, 0) and the cylindrical inclusions reside within a space of 4–7 mm underneath the implant (i.e., BIC) (Figure 4a, bottom). Figure 4b shows the *B*_1_^+^ vector component that rotates in the same direction as the transverse magnetization, (Equation (1) in Note S2, Supporting Information, based on in‐plane components of the *B*_1_ field)^[^ ^43^ ^]^ for the case with and without the implant, plotted at the *xy*‐plane that intersects the center of the inductor coil in the BIC (0, 0, 3.5). The *B*_1_^+^ locally increases in an area of ≈ 50 mm^2^ surrounding the BIC from an average of 3–7 µT, reaching a maximum at 12.3 µT at the center of the single loop inductor in the BIC.

The SNR of the coil follows a commonly used linear scaling relationship^[^ ^44^, ^45^ ^]^ with respect to the mean magnetic field inside the coil according to $\mathrm{SNR}\propto \frac{B_{\mathrm{mean}}}{\sqrt{R}}$, where *B*_mean_ is the mean magnetic field over the total volume of the imaging sample (80 cm^3^ for the phantom in Figure 4) and *R* is the resistance from the sample and the RF coil. The complete expression of the SNR calculation is given by Equation (3) in Note S2, Supporting Information, as a ratio between the magnetic vector fields and the square root of the power absorbed by the tissue phantom.^[^ ^43^, ^45^ ^]^ Figure 4c shows the spatial (*yz*‐plane, the implant located at *y* = 0) enhancement of the SNR over an area of 40 mm × 40 mm in the phantom. Compared to the case without the BIC, the maximum value of the SNR with the implant increases by a factor of ≈10 and ≈2 at the positions of 1 mm (*y* = −1) of 7 mm (*y* = −7), respectively, simulated in the region of interest (ROI, 10 mm radius) directly underneath the implant at the cylindrical inclusions.

The profile of SNR away from the BIC decays with the inverse cube of the depth position as expected for single loop geometries.^[^ ^46^ ^]^ Similarly, Figure 4d presents the bell‐shaped curves of SNR at different implantation depths as a function of the lateral position in the *xy*‐plane. Here, the coordinate of (0, 0, 0) corresponds to the center of the inductor. The simulated distribution of SNR in the *xz*‐plane has maxima at locations directly underneath the implant. The SNR scale enhances with decreasing implant depth over the *x* = −5 to *x* = 5 mm (ROI, 10 mm radius). For *y* = 1, the largest signal occurs as shown in the two peaks at *x* = −3.5 and *x* = 3.5 mm, due to the conductive edges of the loop inductor.

**Figure 5:**
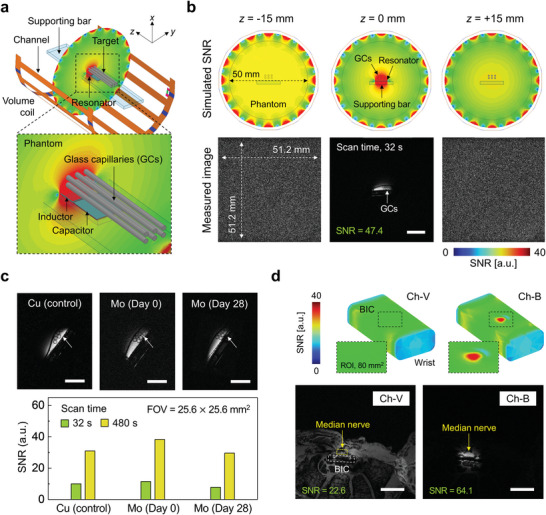
Simulated and experimental results for imaging capabilities enabled by a BIC. a) Schematic illustration of the configuration of a volume coil (63 mm diameter) and a phantom used for MRI. The volume coil has 16 channels, and the phantom consists of a rectangular bar as a support for the BIC and six glass capillaries (800 µm diameter) as an imaging target. Scale bar, 10 mm. b) Measured images with the phantom at three different positions along the *z*‐axis (*z* = 0 and *z* = ±15 mm) (bottom). Corresponding simulated distributions of SNR (top). FOV: 51.2 × 51.2 mm^2^; Scan time: 32 s; SNR in ROI: 47.4. c) Images acquired from the phantom with different resonant devices (top); Cu (non‐bioresorbable device as a control), Mo on Day 0 (bioresorbable device), and Mo on Day 28 (bioresorbable device). Corresponding SNRs in ROI of each device with a scan time 32 s (green) and 480 s (yellow) (bottom). Each white arrow indicates 6 GCs. FOV: 25.6 × 25.6 mm^2^. Scale bars, 5 mm. d) Measured Ch‐B and Ch‐V images of cadaver wrist (bottom). Corresponding simulated distribution in SNR at the skin surface above the BIC (top). FOV: 51.2 × 51.2 mm^2^; Scan time: 32 s; SNR in ROI: 22.6 for Ch‐V and 64.1 for Ch‐B. All scale bars, 10 mm.

Implanting such BICs in experimental phantoms and in a human cadaver arm leads to strong enhancements in the MR signal within an adjacent field of view (FOV, size of the area being imaged). **Figure** **5**a shows the setup for these studies, where the top half of the birdcage is hidden for visualization purposes. The phantom consists of a PBS filled cylinder (50 mm diameter) with a rectangular bar to support a BIC and an adjacent bundle of six glass capillaries (GCs, 800 µm diameter). A cylindrical, quadrature RF volume coil with 16 channels, (63 mm diameter) allows for loading this phantom inside and rotating the BIC to primarily couple with the one channel of the volume coil (Figure S13a,b, Supporting Information). Images (bottom row) of three slices acquired at three positions along the *z*‐axis and corresponding FEA (top row) results demonstrate that the presence of the BIC significantly enhances the signal strength around the device (Figure 5b; Figures S13c and S14, Supporting Information).

Under inductive coupling between the BIC and one channel in the volume coil, referred to in the following as Ch‐B (and not coupled as Ch‐V), the image of a central slice (0, 0, 0) shows a distinct cross‐sectional view of the six GCs compared to two outer slices (0, 0, ±15), which show only noise because they are out of the RF sensitivity of the BIC. Furthermore, from the large FOV images (51.2 × 51.2 mm^2^; scan time, 32 s), the SNRs measured within an ROI adjacent to the GCs are 47.4 for the Ch‐B image and 11.3 for the Ch‐V image (Figure S14, Supporting Information), respectively. The effect of the differences between the susceptibility of Mo and the tissue on MRI is negligible, for cases where the gap between the coil and the tissue is several times greater than the radius of the conductor.

Changes in voxel size and scan time affect the SNR (Figure 5c). The six GCs images scanned with a BIC (FOV, 25.6 × 25.6 mm^2^; scan time, 32 s) have the voxel size of 1/4 of that of the image scanned with FOV of 51.2 × 51.2 mm^2^ (Figure 5b), thereby resulting in four times lower SNR (11.5). The Mo device serves as a bioresorbable alternative to a copper (Cu) device with similar SNR values and imaging performance, despite greater resistance of Mo compared to with Cu. This result indicates that the effective resistance of the imaging sample is a dominant source of noise in these experiments. On day 28 after soaking BIC in PBS, the SNR of the image acquired through this BIC presents a slight decrease likely due to hydration of the device causing a reduction in the resonant frequency. Nevertheless, the targeted six GCs are still clearly visible. The device performance remains adequate, with some slight degradation, for ≈30 days. The constituent materials undergo bioresorption subsequently, over timescales determined by their chemistries, as shown in Figure 1d.

As an example of a clinical use case, a quantitative MRI evaluation of a median nerve typically involves an in‐plane resolution of 1.5×1.5 mm^2^,^[^ ^47^ ^]^ for a global analysis of the nerve in cross section. Individual fascicles of the median nerve (and other peripheral nerves) are <1 mm in diameter. Thus, improvements in resolution to reach these length scales would enable greater specificity about the extent of regeneration (or not) across the full collection of fascicles within the nerve. The BIC demonstration here shows an SNR increase of four times, which alone enables voxels of equal to one half of the volume possible without enhancement, for an in plane resolution of ≈1 mm × 1 mm. In practice, the improvements may reach levels as high as an order of magnitude. Another benefit of the BIC is to enable a much‐reduced field of view, and further increase in the SNR by allowing faster scanning and more signal averaging, or lower bandwidth/lower echo time single‐shot imaging. Figure 5d shows practical MR images (bottom) from the wrist of human cadaver and corresponding simulation with a simplified 3D model of the human wrist (top and Figure S15, Supporting Information), where the BIC is implanted 8 mm underneath the surface of skin and adjacent to the median nerve. Consistent with the results in the phantom tests, the BIC increases the SNR measured in an ROI defined within the median nerve through the interaction with the volume coil (i.e., inductive coupling; Ch‐B), as compared to direct imaging with the volume coil (Ch‐V) and as demonstrated in simulation. These results provide a simple example of the potential use of a BIC in human tissue, although the benefit compared to use of a surface coil will be greater for deeper nerve locations.

## 3 Conclusion

The material compositions, fabrication strategies, and design layouts introduced here provide the foundations for devices with performance characteristics that can improve the capabilities of advanced MRI methods. Here, small, implantable *LC*‐resonant circuits enable enhanced imaging over small regions of interest. The key unique feature of this bioelectronics technology is the use of bioresorbable materials and architectures that allow stable operation over a clinically relevant period of time, followed by complete dissolution into surrounding biofluids. The result provides significant increases in the SNR for imaging of deep tissues, without the need for subsequent surgical extraction. The biocompatible, natural oil layers in the device construction are critically important as effective temporary barriers to biofluid penetration, to prevent drift in the resonant frequencies during an operating timeframe. Demonstrations of use in imaging of a phantom nerve structure and tissues from a human cadaver indicate excellent performance and reliable operation for 28 days.

## 4 Experimental Section

### Fabricating the BICs

The fabrication began with spin‐coating (4000 rpm for 1 min) of a solution of PVA (*M*_w_ 13 000–23 000, Sigma‐Aldrich; 20 wt% in water) on a 50 µm thick Mo foil, followed by laser cutting (ProtoLaser U4, LPKF Laser & Electronics) into an electrically connected pattern composed of one planar coil (square layout, 1 turn, 7 mm wide, 7 mm long, 200 µm in trace width) and two plates (≈42 mm^2^ area for each) for the inductor and capacitor, respectively. Overlapping the plates allowed insertion of a PLGA (65:35 [lactide:glycolide], *M*_w_ 40 000–75 000, Sigma‐Aldrich) film (35 µm thick) in between. The PLGA film, bonded to these plates by hot‐pressing (≈70 °C) for 2 min, to form an insulating layer in a parallel plate capacitor configuration. Embedding the entire sample into an encapsulating structure of PA or OPA completed the fabrication process.

### Encapsulating the Devices

PA and OPA served as encapsulating materials. The synthesis of PA involved mixing 4‐pentaonic anhydride (monomer I), 1,3,5‐Triallyl‐1,3,5‐triazine‐2,4,6(1H,3H,5H)‐trione (monomer II), 1,4‐butanedithiol (crosslinking reagent), and 2,2‐dimethoxy‐2‐phenylacetophenone (photoinitiator, total mass of 0.5%). A silane (trichloro(1H, 1H, 2H, 2H‐perfluorooctyl) silane) coated mold of polydimethylsiloxane (PDMS) defined uniform films with well‐defined thicknesses via first infiltrating the precursor liquid into the gap between the mold and a glass substrate^[^ ^33^ ^]^ and then exposing the material to ultraviolet light (590 µWcm^−2^ intensity, 365 nm wavelength) for 5 min to yield a solid with a tacky surface for putting on the BIC device. Repeating these steps with another mold but with an exposure time of 10 min completed the formation of a fully sealed encapsulating structure. For the case of OPA, sequential repetition of these molding procedure with oil and PA enabled the formation of bilayer structure of OPA (See Figure S6 for details, Supporting Information). Before implantation, each device was immersed in 1 × PBS (pH 7.4 at 37 °C) to stabilize the resonant frequency to ≈200 MHz. All materials obtained from Sigma‐Aldrich were used as received.

### Measuring the Resonant Frequencies

The readout system consisted of a planar coil (circular layout, 1 turn, 25 mm diameter, 1.5 mm trace width) connected to a network analyzer (E5062A, Agilent Technologies). The network analyzer measured the S‐parameter (S_11_ element), including the real (ReS_11_) and imaginary (ImS_11_) parts, in reflective mode. These S_11_ values allowed determination of the real part of the impedance (Re *Z*) according to:

(1) ReZ=Z01−ReS112−ImS1121−ReS112+ImS112

where *Z*_0_ is 50 Ω as the real impedance of the network analyzer.

### Testing the Water Permeability

Electron beam evaporation (AJA Orion 8 evaporation system, AJA International Inc.) formed a Mg film with thickness of 300 nm on an oxidized silicon substrate. Photolithographically patterning a layer of photoresist (AZ5214, MicroChemicals) followed by a wet etching process (etchant: a mixed solution of acetic acid and deionized water in a volume ratio of 1:10) defined serpentine traces of Mg as the resistor test structures. Deposition of films of gold at the ends of these traces defined pads for electrical connection. Covering these Mg resistors with layers of PA (135 µm thick) and OPA (oil: 350 µm thick, PA: 135 µm thick) and then placing a well structure formed with PDMS and filled with 1 mL of 1 × PBS (pH 7.4 at 37 °C) on top yielded a setup for testing water permeation through these encapsulating structures. Refreshing the PBS once per day after each measurement minimized the effect of build‐up of reaction products in the PBS.

### In Vitro Testing Degradation of BICs

The tests began with immersion of devices in plastic well chambers (37 mm diameter) filled with 7 mL of 1 × PBS (pH 7.4) at 37 °C. A network analyzer recorded changes in the resonant properties of the devices once per day for 30 days. Refreshing the PBS after each measurement minimized the effect of build‐up of reaction products.

### Reactive Diffusion Modeling

See Note S1, Supporting Information, for details.

### Electromagnetic simulations

See Note S2, Supporting Information, for details.

### MRI Validation and Data Analysis

Imaging phantom–A cylindrical (50 mm diameter) plastic phantom filled with 1 × PBS and 1 mm, of MRI contrast agent (Gd‐DTPA) held a BIC (or non‐resorbable Cu version) and a bundle of 6 GCs (1 mm outer diameter; 0.8 mm inner diameter). A 4.7 T MRI system (Varian/Agilent direct drive console) using a RF volume coil (63 mm diameter) allowed imaging of this phantom. Rotation of the phantom within the volume coil maximized the signal on one linear channel (coupled to the BIC, Ch‐B) and minimized the signal on the other (coupled to the volume coil, Ch‐V). During the experiments, the GCs were positioned off‐center from the coil to allow a uniform signal region for calculation of a mean image intensity. For both the Cu and Mo experiments, the cluster of glass cylinders were simply taped to the supporting bar–no effort was made to match the exact arrangement of GCs.

Use of a rapid acquisition with relaxation enhancement (aka, RARE) protocol (sequence, 256 × 256 sampling matrix; echo spacing, 11 ms; echo train length, 8; repetition time [TR], 500 ms) yielded a collection of phantom images. Images with the BIC were repeatedly acquired from three 1 mm‐thick slices spaced 15 mm apart and centered on the BIC, with using both channels (Ch‐B and Ch‐V) and two field of views (25.6 × 25.6 mm^2^ and 51.2 × 51.2 mm^2^). *M*_signal_ and *M*_noise_ defined the mean intensity of image measured from both a signal and a noise region of interest (ROI), respectively, thereby leading to *M*_signal_/(*M*_noise_/$\sqrt{\pi /2}$) as equal to image SNR. The signal ROI for Ch‐B and Ch‐V images extended over the bright region in the middle slice, adjacent to the glass capillaries and the BIC, and from the corresponding region in the slice offset at −15 mm, respectively. Imaging cadaver—A demonstration of the operation of a BIC in human tissue involved implantation directly beneath the median nerve in the carpal tunnel region of the wrist of a cadaver arm. Imaging used the same MRI systems as for phantom scans described above, with a volume coil with a diameter of 144 mm. As above, the Ch‐B and Ch‐V acquired the image from a 1 mm‐thick slice centered on the BIC with a FOV of 51.2 × 51.2 mm^2^ (spoiled gradient echo protocol, echo time, 6 ms; TR, 50 ms; flip angle, ≈15°; and 32 averaged excitations). For images from both channels, the ROI within the median nerve provided the SNR values.^[^ ^47^ ^]^

### Processing Cadaveric Specimen

The fresh‐frozen, cadaveric forearm (fingertip to elbow) was obtained from the United Tissue Network (UTN) (Phoenix, AZ, USA; American Association of Tissue Banks; accreditation #00220/3). Informed written consent was obtained from the potential donor to donate their body. The donor and family were supplied with information about policies and procedures that will take place after the potential donor is deceased (and the sample used in the test was fully de‐identified prior to the receipt from the UTN). The cadaveric forearm was stored in PBS at −20 °C and thawed to room temperature for at least 24 h before undergoing further processing, including dissection for biomechanical implantation of the BIC and subsequent MR imaging.

### Statistical Analysis

Excel (Microsoft) calculated average values and error bars. All error bars in the plots including supporting information correspond to standard deviations. Each data point in Figure 2 represents the average measured from independent experiments and samples (*n* = 3). In Figure 5, a single device for each experiment yielded imaging multiple times with similar results.

## Conflict of Interest

The authors declare no conflict of interest.

## Supporting information

Supporting Information

## Data Availability

The data that support the findings of this study are available in the supplementary material of this article.
