# Supplementary material for: Implantable, Bioresorbable Radio Frequency Resonant Circuits for Magnetic Resonance Imaging
Source: Adv Sci (Weinh). 2023 Jun 25;11(27):2301232. doi: 10.1002/advs.202301232 (PMC11251549; doi:10.1002/advs.202301232)
Supplement: Supplementary file 1 — Supporting Information [file ADVS-11-2301232-s001.pdf]

ADVANCED SCIENCE Open Access

Supporting Information

for *Adv. Sci.*, DOI 10.1002/advs.202301232

Implantable, Bioresorbable Radio Frequency Resonant Circuits for Magnetic Resonance Imaging

Geumbee Lee, Mark D. Does, Raudel Avila, Juyeon Kang, Kevin D. Harkins, Yunyun Wu, William E. Banks, Minsu Park, Di Lu, Xinqiang Yan, Jong Uk Kim, Sang Min Won, Adam G. Evans, Jeremy T. Joseph, Christopher L. Kalmar, Alonda C. Pollins, Huseyin Karagoz, Wesley P. Thayer, Yonggang Huang and John A. Rogers**

ADVANCED SCIENCE
Open Access

Supporting Information

for *Adv. Sci.*, DOI 10.1002/advs.202301232

Implantable, Bioresorbable Radio Frequency Resonant Circuits for Magnetic Resonance Imaging

Geumbee Lee, Mark D. Does, Raudel Avila, Juyeon Kang, Kevin D. Harkins, Yunyun Wu, William E. Banks, Minsu Park, Di Lu, Xinqiang Yan, Jong Uk Kim, Sang Min Won, Adam G. Evans, Jeremy T. Joseph, Christopher L. Kalmar, Alonda C. Pollins, Huseyin Karagoz, Wesley P. Thayer, Yonggang Huang and John A. Rogers**

pporting Information

Implantable, Bioresorbable Radio Frequency Resonant Circuits for Magnetic Resonance Imaging

Geumbee Lee, Mark D. Does, Raudel Avila, Juyeon Kang, Kevin D. Harkins, Yunyun Wu, William E. Banks, Minsu Park, Di Lu, Xinqiang Yan, Jong Uk Kim, Sang Min Won, Adam G. Evans, Jeremy T. Joseph, Christopher L. Kalmar, Alonda C. Pollins, Huseyin Karagoz, Wesley P. Thayer, Yonggang Huang, John A. Rogers**

Note S1. | Reactive diffusion model for PA- and OPA-encapsulated Mg.

A 1D model can be used to analytically solve the governing reaction and diffusion process in the polymer substrate (PA), water-barrier (oil), and encapsulated conductive elements (Mg), while immersed in an aqueous solution (1×PBS, pH 7.4) at body temperature. The model, adopted from the encapsulation strategy for transient electronics described by Li *et al.*[1] and modified in Choi. *et al*[2] for polyanhydrides, is used since the PA/oil/Mg specimen thickness (h_{Mg} + h_{Oil} + h_{PA}), where h_{Mg}, h_{Oil}, and h_{PA} are the initial thicknesses of the Mg, the oil, and PA layers, respectively, are much smaller as compared to the lateral dimensions. The effect of the water-barrier was captured by calculating the change in electrical resistance for different encapsulation labeled as PA (h_{PA}, 135 µm thick) and OPA (h_{Oil} + h_{PA}, 350/135 µm thick). The electrical resistance of Mg layer is given by $R = \frac{R_0 h_0}{h}$, where R_0 is the initial resistance. The analytical equation for normalized Mg thickness as a function of time is given by:

$$\frac{h(t)}{h_0} = 1 - \frac{w_0 M_{\mathrm{Mg}}}{q\rho_{\mathrm{Mg}} M_{H_2O}} k \left[\frac{1}{\sqrt{\frac{D_{Mg} k h_{OPA}^2}{D_{OPA}^2}} \sinh\sqrt{\frac{k h_{Mg}^2}{D_{Mg}}} + \cosh\sqrt{\frac{k h_{Mg}^2}{D_{Mg}}}} t \frac{\sinh\sqrt{\frac{k h_{Mg}^2}{D_{Mg}}}}{\sqrt{\frac{k h_{Mg}^2}{D_{Mg}}}} + \sum_{n=1} \frac{C_n}{\lambda_n}\left(1 - e^{-\lambda_n t}\right) \frac{\sin\left(\sqrt{\frac{\lambda_n - k}{D_{Mg}} h_0^2}\right)}{\sqrt{\frac{\lambda_n - k}{D_{Mg}} h_{Mg}^2}} \sin\left(\sqrt{\frac{\lambda_n}{D_{OPA}} h_{OPA}^2}\right) \right]$$

where, w_0 is the initial water concentration, D_{Mg} is the diffusivity, k is reaction constant of water in Mg and D_{OPA} is the diffusivity of double encapsulation of oil and PA. M_{Mg} and M_{H_2O} are the molecular mass of Mg and water, respectively, and $q = 2$ means the two water molecules that react with each Mg atom in the process and ρ_{Mg} is the density of Mg. C_n is a coefficient obtained from the initial boundary conditions and λ_n are eigenvalues in the water concentration function. The complete details of the analytical model derivation are presented in Li. *et al.*[1] The critical time t_c (i.e., functional lifetime) can be determined when the value of the resistance reaches a critical value (i.e., $R = 500\ \Omega$) and by using $R_0 \approx 45\ \Omega$, measured from experiments, and setting $\frac{h(t_c)}{h_0} = 0.1$. The material parameters used in the analytical model are M_{Mg} = 24 g mol $^{-1}$, M_{H_2O} = 18 g mol $^{-1}$, ρ_{Mg} = 1.738 g cm^{-3}, w_0 = 1 g cm^{-3}, k = 1.2×10^{-3} s^{-1}, D = 6.0×10^{-16} m^2 s^{-1}, and D_{OPA} = 2.55×10^{-15} m^2 s^{-1}.

Note S2. | Electromagnetic simulation: B_1 and SNR

The commercial software ANSYS HFSS was used to perform 3D electromagnetic finite element analysis of two RF birdcage coil geometries at 4.7 T (Larmor Frequency, 200 MHz) to produce a homogeneous and circular polarized magnetic field B_1, perpendicular to B_0, and quantify the SNR in different implantable scenarios. The birdcage coils include two circular loops (i.e., end rings), evenly spaced for an (1) 8-element (210 mm long, 144 mm diameter) and a (2) 16-element conductive (80 mm long, 63 mm diameter) paths (legs), modeled as 2D finite conductive elements, joined by matching capacitors in a low-pass configuration to tune the coils to a the Larmor frequency of 200 MHz.[3] A lumped circuit model was used to determine the value of the matching capacitors C at the end rings by considering the self-inductance of the conductive leg paths and end rings, and the corresponding mutual inductances between the legs. The matching capacitors are 23 pF and 104 pF for the 8-element and 16-element legs birdcage coils, respectively. A cylindrical shield with finite conductivity was included in the modeling. Adaptive meshing is used to refine the mesh and ensure convergence of the simulation. The total number of elements in the FEA model is ~400,000. A schematic of the geometrical dimensions of the birdcage is shown in Figure S11.

To produce the rotational magnetic field B_1, a four-port excitation was adopted through lumped ports placed 90° degrees apart with the phase excitations of 0 °, -90°, 180° and 90° in the end rings resulting in a sinusoidal current distribution in the birdcage legs. B_1^+ is the vector component that rotates in the same direction as nuclei inducing a flip angle with respect to the stationary magnetic field B_0 and B_1^- is the vector component that rotates in the opposite direction defined as[4]

$$B_1^+ = \frac{\left(B_x + iB_y\right)}{2} \tag{1}$$

$$B_1^- = \frac{\left(B_x - iB_y\right)^*}{2} \tag{2}$$

where B_x and B_y are complex in-plane magnetic fields magnitudes in the birdcage, i is the imaginary unit and the asterisk indicates the complex conjugate. The flip angle is defined as $\alpha_n = B_{1n}^+\gamma\tau$ where B_{1n}^+ is the value of B_1^+ in a mesh element in the region of interest, γ is the gyromagnetic ratio of ^{1}H and τ is the duration of the RF pulses.

The SNR in the regions of interest is calculated as[4]

$$\mathrm{SNR} \propto \frac{f^2}{N_{elements}\sqrt{P_{abs}}} \sum_{N_{elements}} \left|W_n(\sin\alpha_n)B_{1n}^-\right| \tag{3}$$

where, f is the Larmor frequency, $N_{elements}$ is the number of mesh elements in the region of interest, W_n is the water content (by percent mass) in the tissues, and B_{1n}^- is the value of B_1^- in a mesh element in the region of interest. P_{abs} is the total power absorbed in the tissue model and is calculated from the magnitudes of the electric field E intensity as,[5]

$$P_{abs} = \frac{1}{2} \sum_{N_{elements}} \left(\sigma_x E_x^2 + \sigma_y E_y^2 + \sigma_z E_z^2\right) \Delta_x \Delta_y \Delta_z \tag{4}$$

where σ is the electrical conductivity of the tissue and $\Delta_x, \Delta_y, \Delta_z$ are the dimensions of the meshed elements in the three principal directions. The values of the relative permittivity, electrical conductivity, and density used in the simulation for the tissue equivalent models with nerve bundles, wrist model, and the bioresorbable implant are shown in Table S1.[4,6]

References

[1] R. Li, H. Cheng, Y. Su, S.-W. Hwang, L. Yin, H. Tao, M. A. Brenckle, D.-H. Kim, F. G. Omenetto, J. A. Rogers, Y. Huang, *Adv. Funct. Mater.* **2013**, *23*, 3106.

[2] Y. S. Choi, J. Koo, Y. J. Lee, G. Lee, R. Avila, H. Ying, J. Reeder, L. Hambitzer, K. Im, J. Kim, K.-M. Lee, J. Cheng, Y. Huang, S. K. Kang, J. A. Rogers, *Adv. Funct. Mater.* **2020**, *30*, 2000941.

[3] G. Giovannetti, L. Landini, M. F. Santarelli, V. Positano, *Magn. Reson. Mater. Physics, Biol. Med.* **2002**, *15*, 36.

[4] W. Liu, C. M. Collins, M. B. Smith, *Appl. Magn. Reson.* **2005**, *29*, 5.

[5] C. Wang, G. X. Shen, *J. Magn. Reson. Imaging* **2006**, *24*, 439.

[6] C. Gabriel, Compilation of the Dielectric Properties of Body Tissues at RF and Microwave Frequencies, Occupational and environmental health directorate, Radiofrequency Radiation Division, *Brooks Air Force Base* **1996**.

Material and tissue	Relative permittivity (1)	Electrical conductivity (S/m)	Density (kg/m^3)
Mo	1	1.76×10^7	10220
PLGA	2.6-2.8	0	1250
PA	4	0	1100
Oil	3.23	0	920
Skin	55.7	0.58	1100
Fat	12.2	0.07	911
Muscle	60.2	0.74	1090
Bone	24.4	0.20	1200
Nerves	39.7	0.38	1075

Table S1. | Parameters of relative permittivity, electrical conductivity, and density.

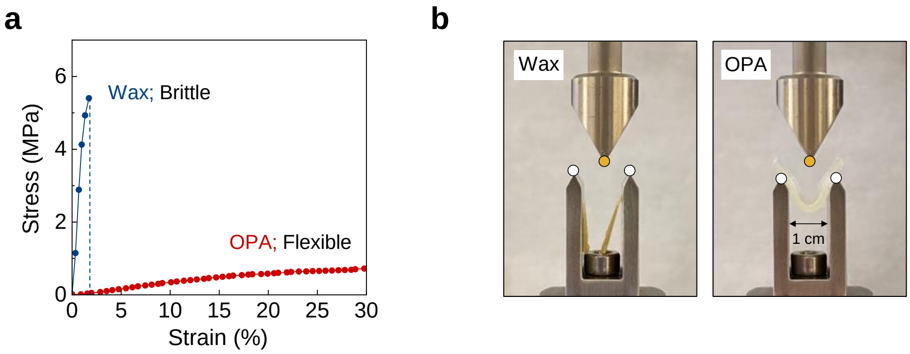

Figure S1. | Mechanical flexibility of wax and OPA layer.

a) Stress-strain curves of the wax (350 μm) and the OPA layer (oil: 350 μm thick; total 600 μm thick) during 3-point flexural test at body temperature (37°C). b) Images showing the brittle wax and the flexible OPA layer after 3-point flexural test. White dots, supporting pins; Yellow dots, loading pins.

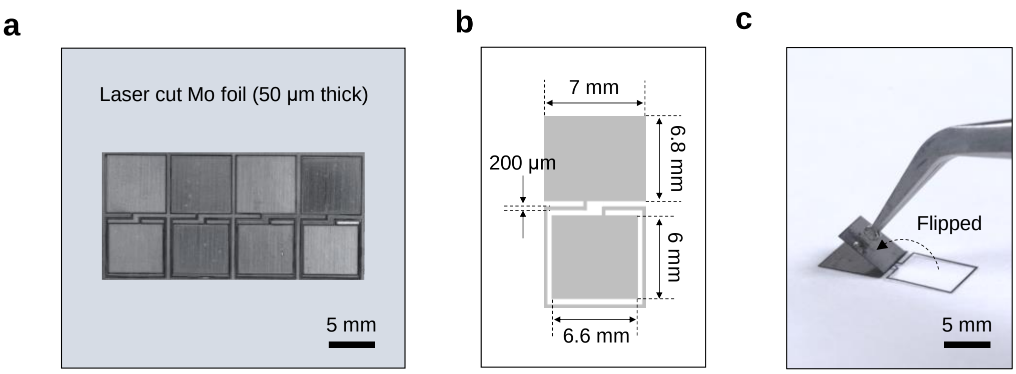

Figure S2. | Details associated with fabrication of a BIC.

a) Laser-cut Mo foil (50 µm thick) as electrodes for a single loop (inductor) and a pair of plates (capacitor). Scale bar, 5 mm. b) Dimensions of a single device. c) Photograph of the flipping process for the metal-insulator-metal structure. Here, a biodegradable layer of PLGA separates two metal plates as an insulating film. Scale bar, 5 mm.

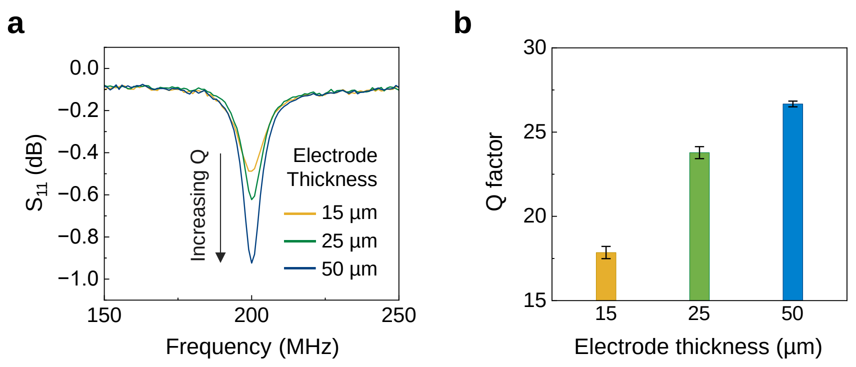

Figure S3. | Radio frequency (RF) behaviors of the BIC with various electrode thicknesses.

a) Measured RF behavior (S_{11}) with the 15, 25 and 50 μm-thick BICs. The resonant frequencies, ≈200 MHz. b) Calculated quality factor (Q-factor) as a function of electrode thickness. Q-factor increases with an increase in the electrode thickness. Independent samples, n=5.

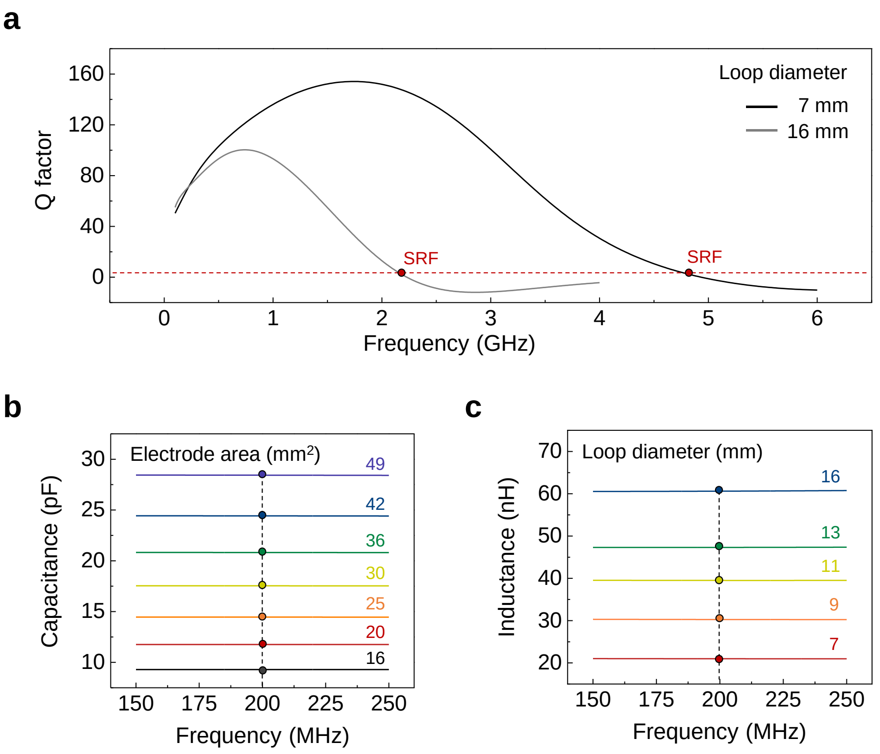

Figure S4. | Simulation results in electrical performance characteristics of the *LC*-resonant circuit.

a) Q-factor as a function of frequency. The zero of Q-factor defines self-resonant frequency (SRF, red dots) for *LC*-resonant devices. b) Simulation results for the capacitance depending on the area of the metal plate as a function of frequency. c) Simulation results for the inductance depending on the diameter of the loop as a function of frequency.

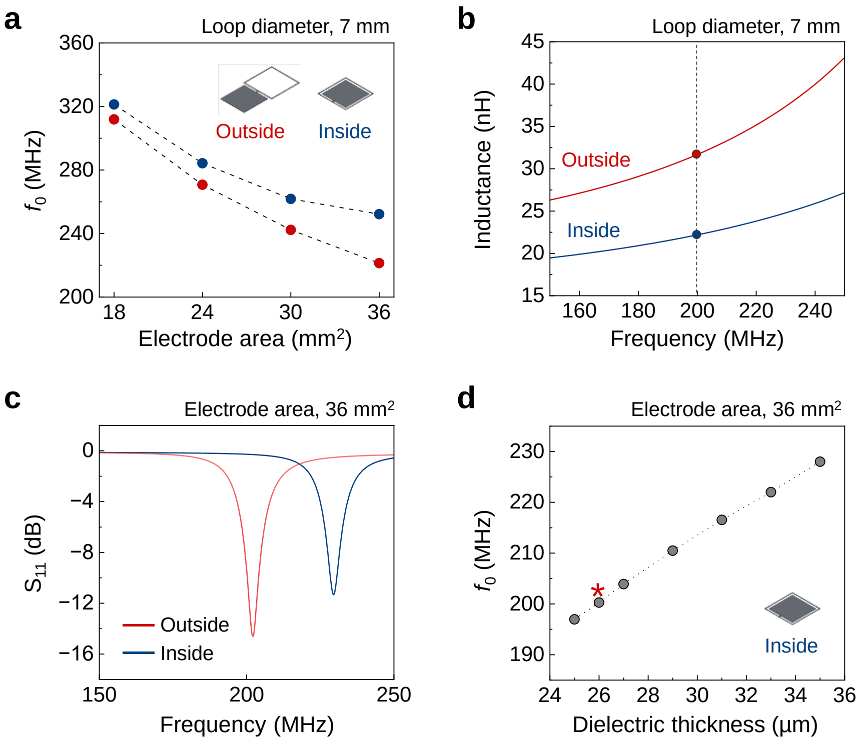

Figure S5. | Simulated electrical characteristics in the *LC*-resonant circuit with different layouts.

a) f_0 as a function of electrode area. b) Inductance as a function of frequency. c) Efficiency of RF behaviors of fully encapsulated devices with different layouts in PBS. d) Change in f_0 depending on the thicknesses of dielectric layers with the inside layout of device. A red asterisk corresponds to optimized thickness for desired f_0 in inside layout (electrode area, 36 mm^2; loop diameter, 7 mm). Two *LC*-resonant circuits used same dimension, but only different layouts. Electrode area, 36 mm^2; thickness in dielectric layer, 35 μm; loop diameter, 7 mm.

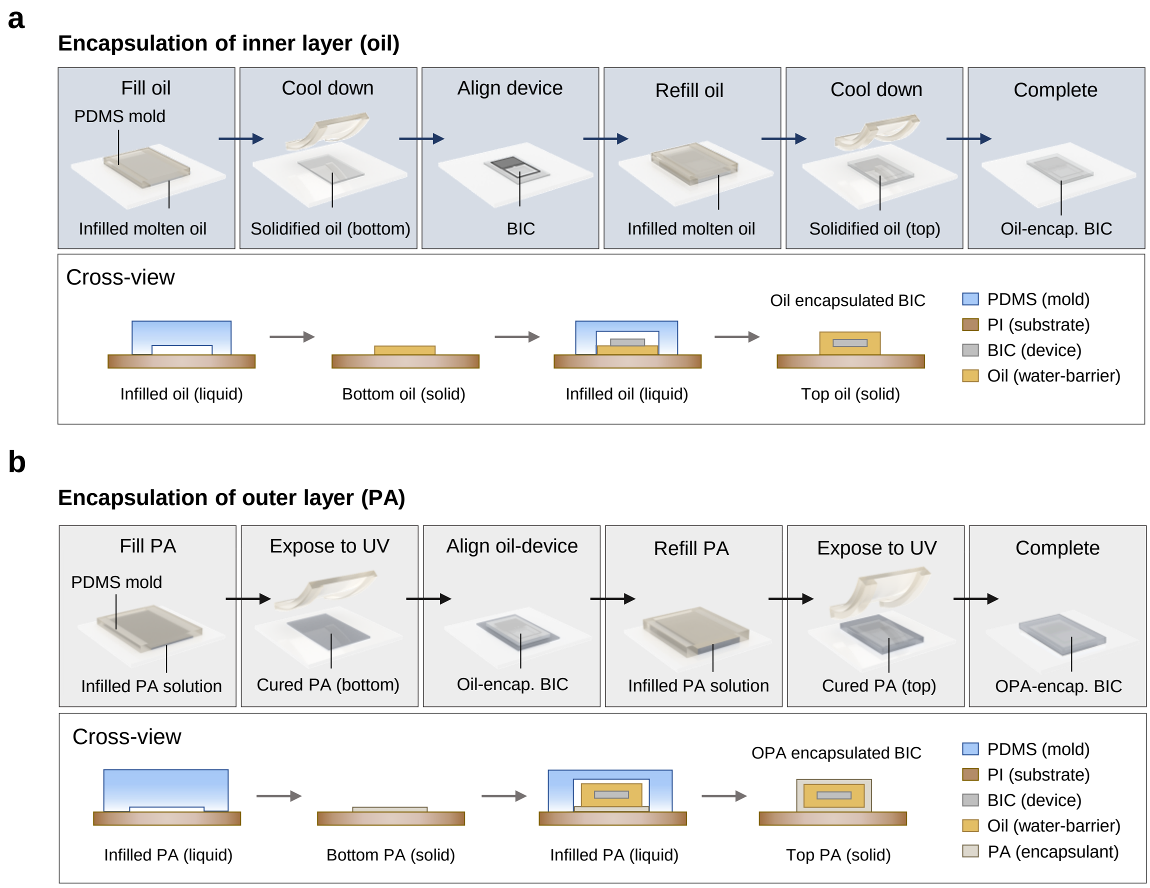

Figure S6. | Schematic illustrations for the bilayer encapsulating strategy.

a) Encapsulation strategy for a water-barrier with oil: Molten oil infills the mold (i.e., silane treated PDMS) by capillary action. After cooling, the solidified oil remains on the temporary substrate, followed by removing the mold. Placing the device on the oil layer, refilling the mold with molten oil and cooling yields a fully, oil-encapsulated device. Bottom, cross-view. b) Encapsulation strategy for the supporting layer with PA: the procedure mentioned above applies to the oil-encapsulated device and liquid mixture of PA, thereby yielding the OPA-encapsulated device. Bottom, cross-view.

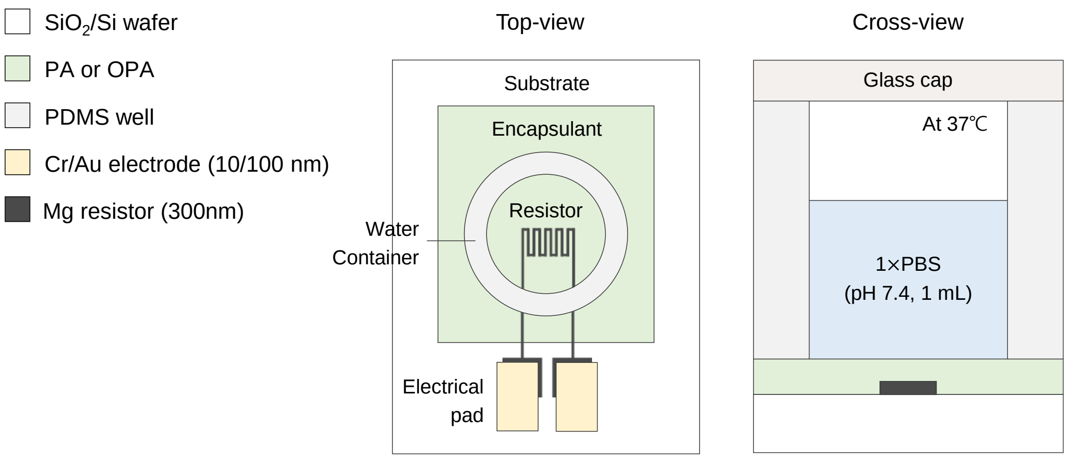

Figure S7. | Experimental setup for water-permeability test.

Schematic illustration of the experimental setup, designed to test water permeability through encapsulants (PA or OPA) with exposure to 1×PBS (pH 7.4 at 37°C).

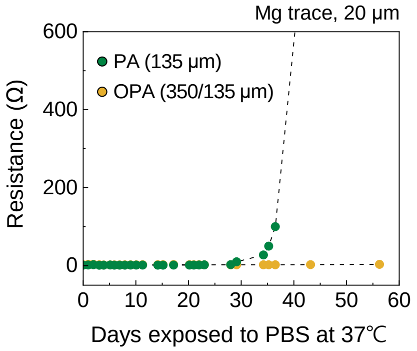

Figure S8. | Water permeability of PA- and OPA-encapsulated Mg traces.

Changes in the resistances of Mg foil traces (≈20 μm thick) encapsulated with PA (135 μm thick) and OPA (oil: 350 μm thick, PA: 135 μm thick) layers during exposure to 1×PBS (pH 7.4) at 37°C.

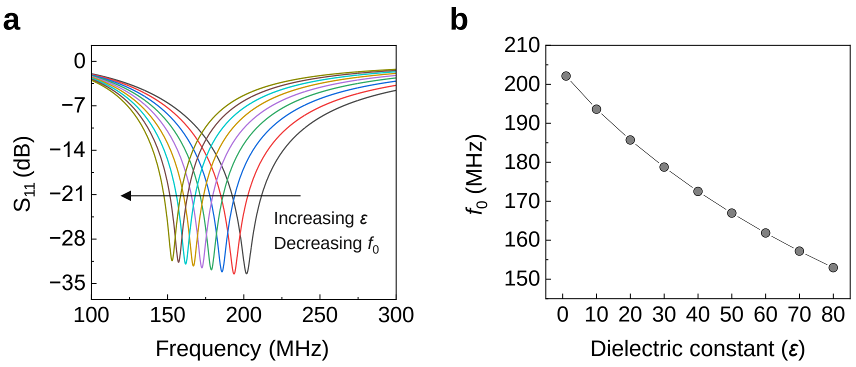

Figure S9. | Simulation results for RF responses of the hydrated BIC.

a) Simulated S_{11} as a function of frequency. Hydration of the device increases the dielectric constant, shifting the resonant frequency (f_0). b) Drift in f_0 depending on the degree of hydration of device.

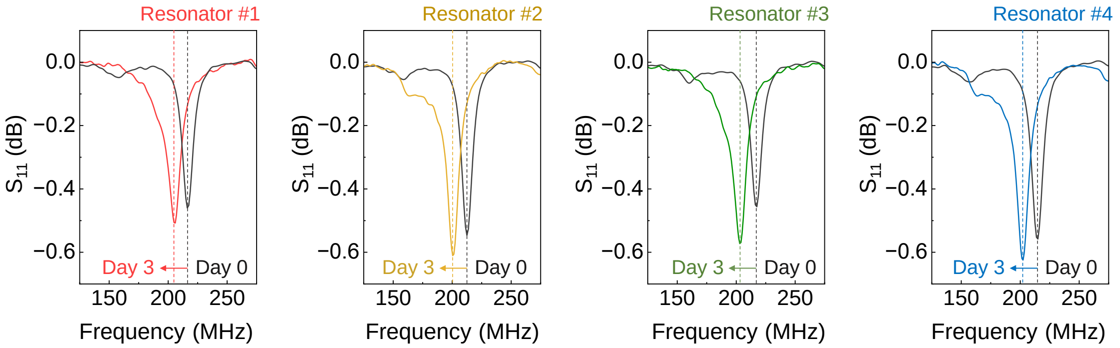

Figure S10. | Evaluation of reproducibility for stabilization of f_0 with individual BIC.

Stabilization process for devices with a f_0 of ≈220 MHz, immersed in 1×PBS (pH 7.4 at 37ºC) before implantation. From day 3, the devices used for testing stabilize at a f_0 of ≈200 MHz. Independent samples, n=4.

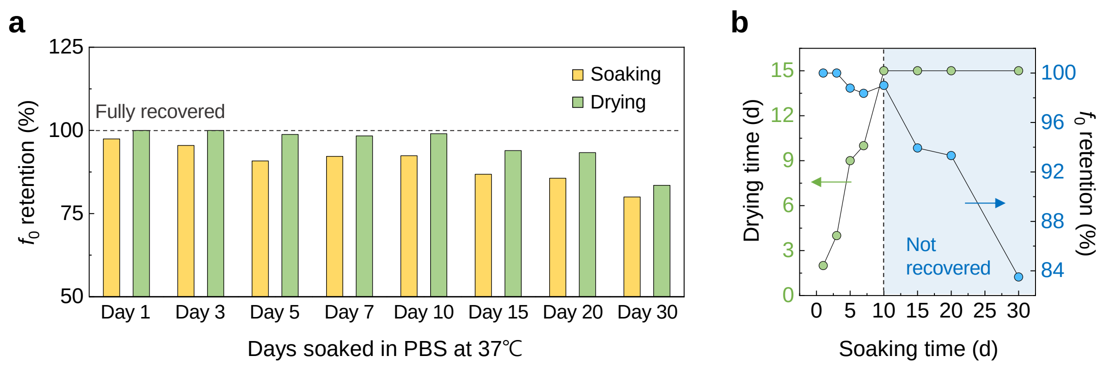

Figure S11. | Evaluation of recovery in f_0 of the hydrated BICs by drying.

a,b) Retention of the f_0 of the resonators after drying. As the soaking time of the devices increases, the drying time also increases to return to the original value of f_0. Long-term immersion may alter the metal features (e.g., oxidation or hydrolysis), resulting in irreversible drift of f_0. Independent samples, n=8.

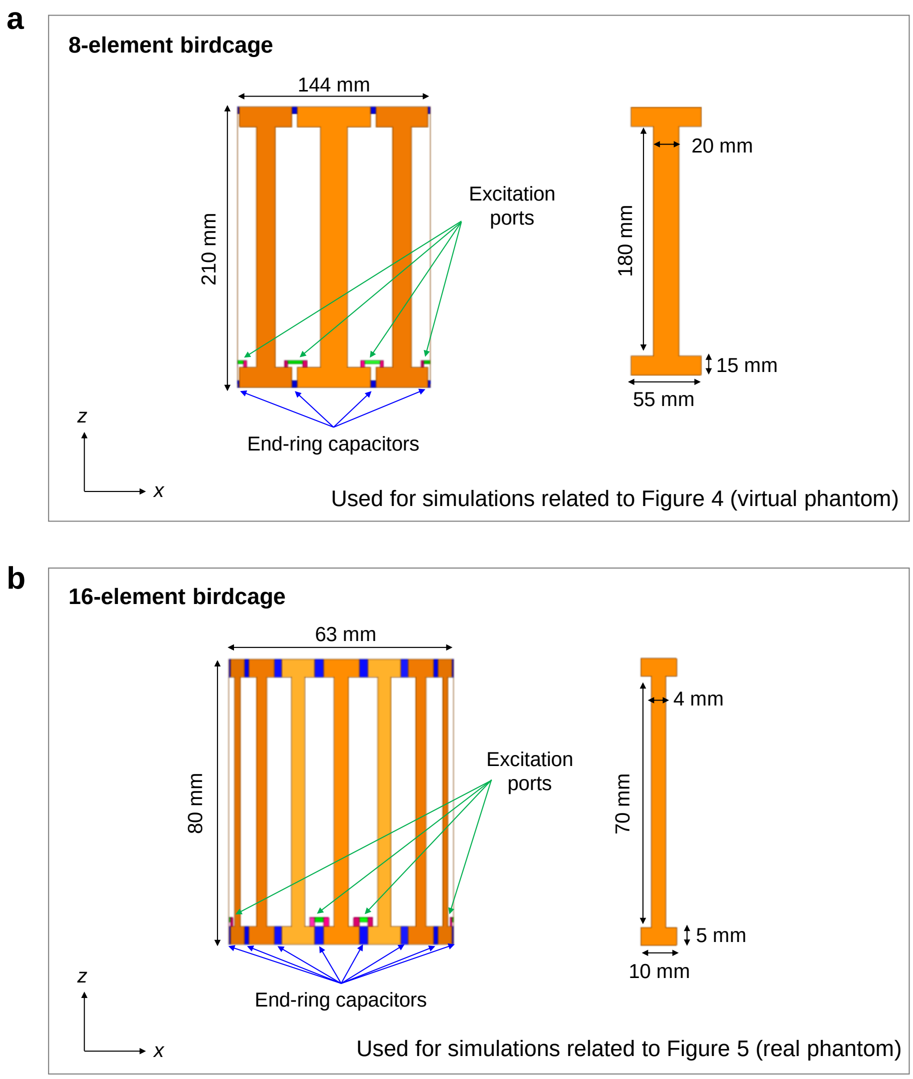

Figure S12. | Geometries and dimensions of birdcage coils used in FEA simulations.

a) An 8-element low-pass birdcage coil (left) and its single leg (right), and b) a 16-element low-pass birdcage coil (left) and its single leg.

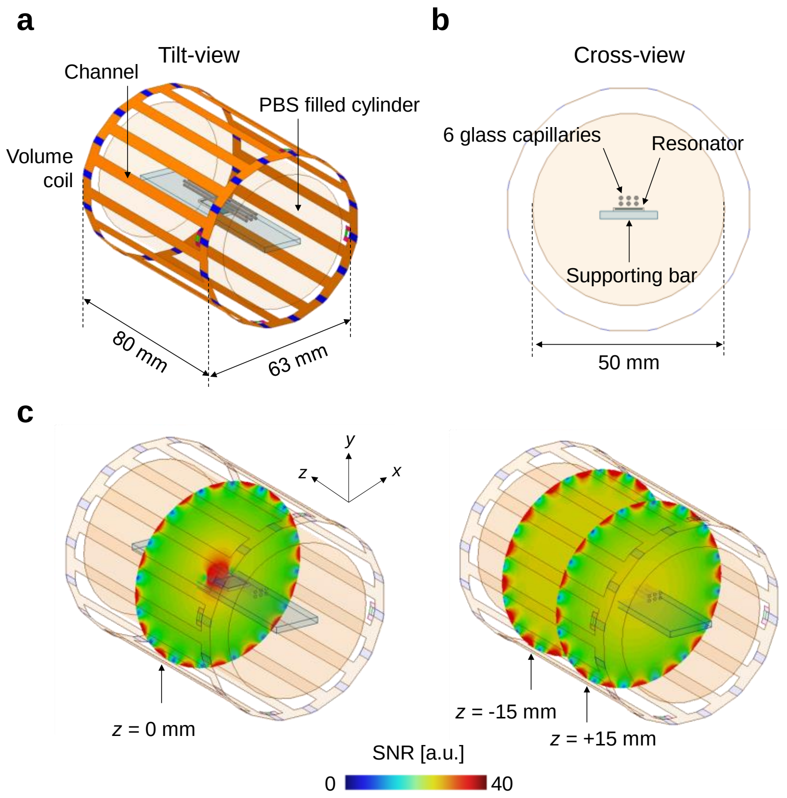

Figure S13. | Geometry and dimension of a volume coil used for imaging and simulation of a phantom.

a) Schematic illustration of configuration of a volume coil with 16 channels. Diameter, 63 mm; length, 80 mm. b) Cross-sectional view of the volume coil loaded with a phantom. Phantom inside a PBS filled cylinder (50 mm inner diameter) consists of 6 glass capillaries (GCs, 1 mm outer diameter; 0.8 mm inner diameter), a BIC and a supporting bar. c) Simulated distributions of SNR at 3 different positions along the *z*-axis (z=0 and z=±15 mm).

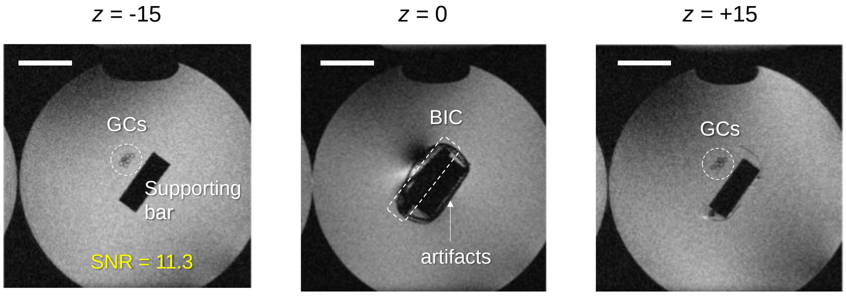

Figure S14. | Phantom images of 3 slices acquired with a volume coil (Ch-V).

At z = 0, the presence of the BIC causes distortion of the static magnetic field when acquiring an image through Ch-V, resulting in artifacts in the images. At z = -15 mm, the MR images clearly show the 6 GCs with SNR of 11.3. Here, none of the channels of the volume coil inductively couple with the BIC. All scale bars, 10 mm.

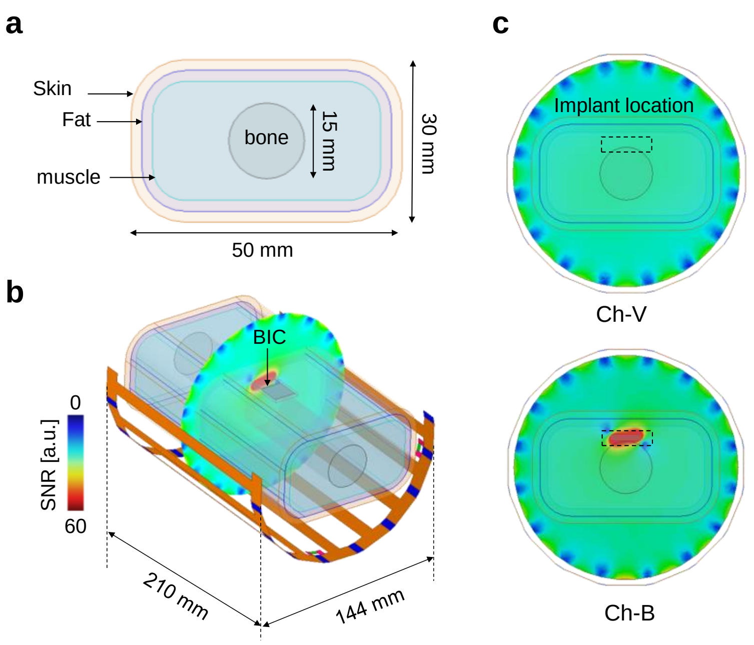

Figure S15. | Specifications of the MRI system used for imaging of a cadaver arm.

a) Cross-sectional view and dimensions of the wrist of a cadaver arm. b) Schematic illustration of the configuration of a volume coil with 8 channels. Outer diameter, 144 mm; length, 210 mm. c) Simulated distributions of SNR near a device implanted to a depth of 8 mm underneath the skin. SNR shows 19.6 and 59.5 with Ch-V and Ch-B, respectively.
